# Poly(HEMA-co-MMA) Hydrogel Scaffold for Tissue Engineering with Controllable Morphology and Mechanical Properties Through Self-Assembly

**DOI:** 10.3390/polym16213014

**Published:** 2024-10-27

**Authors:** Ja-Rok Kim, Yong Sang Cho, Jae-Hong Park, Tae-Hyun Kim

**Affiliations:** 1 R&D Center, TE BioS, Co., Ltd., 194-41, Osongsaengmyeong 1-ro, Heungdeok-gu, Cheongju-si 28160, Republic of Korea; lab21_yhj@tebios.com (J.-R.K.); lab20_pjh@tebios.com (J.-H.P.); 2 Medical Device Development Center, Daegu-Gyeongbuk Medical Innovation Foundation (K-MEDI hub), 80 Cheombok-ro, Dong-gu, Daegu 41061, Republic of Korea; yscho@kmedihub.re.kr

**Keywords:** (2-hydroxyethyl methacrylate), methyl methacrylate, poly(HEMA-co-MMA), hydrogel scaffold, soft-tissue

## Abstract

Poly(2-hydroxyethyl methacrylate) (PHEMA) has been widely used in medical materials for several decades. However, the poor mechanical properties of this material have limited its application in the field of tissue engineering. The purpose of this study was to fabricate a scaffold with suitable mechanical properties and in vitro cell responses for soft tissue by using poly(HEMA-co-MMA) with various concentration ratios of hydroxyethyl methacrylate (HEMA) and methyl methacrylate (MMA). To customize the concentration ratio of HEMA and MMA, the characteristics of the fabricated scaffold with various concentration ratios were investigated through structural morphology, FT-IR, mechanical property, and contact angle analyses. Moreover, in vitro cell responses were observed according to the various concentration ratios of HEMA and MMA. Consequently, various morphologies and pore sizes were observed by changing the HEMA and MMA ratio. The mechanical properties and contact angle of the fabricated scaffolds were measured according to the HEMA and MMA concentration ratio. The results were as follows: compressive maximum stress: 254.24–932.42 KPa; tensile maximum stress: 4.37–30.64 KPa; compressive modulus: 16.14–38.80 KPa; tensile modulus: 0.5–2 KPa; and contact angle: 36.89–74.74°. In terms of the in vitro cell response, the suitable cell adhesion and proliferation of human dermal fibroblast (HDF) cells were observed in the whole scaffold. Therefore, a synthetic hydrogel scaffold with enhanced mechanical properties and suitable fibroblast cell responses could be easily fabricated for use with soft tissue using a specific HEMA and MMA concentration ratio.

## 1. Introduction

Synthetic hydrogel scaffolds are widely used for the reconstruction of damaged tissues by providing a space for cell growth and differentiation. Moreover, these scaffolds enable cells to work in a similar way to those found in vivo by mimicking the natural extracellular matrix (ECM) [1,2]. Synthetic hydrogel scaffolds can exhibit various properties such as biocompatibility [3,4], pore size [5], mechanical modulus [6,7], wettability [8], substrate roughness [9,10], and dimensional stability [11], making them ideal for successful in vivo cell culture.

In recent research, the roughness and stiffness of hydrogel scaffolds were found to be key factors in cell adhesion, cell shape, tissue organization, the surface energy of materials, and wettability [12,13,14]. A. Ranella et al. showed that the spread and viability of cells were affected by the roughness of the scaffold [9]. Thus, various techniques, including 3D printing [15,16,17,18], nano-micro patterning [19,20], the use of nanoparticles [21], and plasma treatment [22], were used for enhancing cell-to-substrate interactions by controlling the roughness of the scaffolds developed in recent research. However, these studies used materials that did not confirm biocompatibility, and a considerable amount of manufacturing time was spent on non-self-assembly. Furthermore, high-stiffness (>1000 Pa) materials such as silicon, polystyrene, and poly ε-caprolactone were used for fabricating the roughness of the scaffolds, which may not be adequate for soft tissue applications that require soft stiffness (100–1000 Pa).

Poly(2-hydroxyethyl methacrylate) (PHEMA), with high biocompatibility, has been widely used in medical materials such as in contact and intraocular lenses [23], artificial corneas [24], vitreous humor replacement [25], artificial emboli [26], and burn dressings and surgery [27]. PHEMA is a di-functional monomer that contains both hydroxy and carboxylic acid. Moreover, it has advantages such as suitable mechanical strength and the ability to supply nutrition or various gasses for cell growth due to its high water uptake. Additionally, PHEMA has a suitable gel fraction and phase separation due to its di-functional properties, which are useful for the fabrication of hydrogels. This provides the possibility of fabricating scaffolds with various roughness and nano/micro-pore sizes through the concentration of solvents, initiators, and crosslinkers to enhance cell adhesion and differentiation [28,29]. Common crosslinking agents used in the polymerization of PHEMA include diacrylates and dimethacrylates, polyfunctional acrylates and methacrylates, and allyl methacrylate (AM) [30,31]. Diacrylates and dimethacrylates, such as ethylene glycol dimethacrylate (EGDMA) and triethylene glycol dimethacrylate (TEGDMA), possess two acrylate or methacrylate groups, enabling them to form crosslinks between HEMA chains [30]. Conversely, crosslinkers such as polyfunctional acrylates and methacrylates, such as pentaerythritol triacrylate (PETA) and pentaerythritol tetra-acrylate (PET), contain multiple acrylate or methacrylate groups, resulting in a denser crosslinked network [32,33]. This higher degree of crosslinking can lead to improved mechanical properties, such as increased modulus and reduced swelling. Additionally, allyl methacrylate (AM) can react with the double bond of HEMA to form a crosslink, providing another option for tailoring the properties of the resulting hydrogel [34]. However, fabrication methods that only use PHEMA are limited in terms of fabricating scaffolds with suitable roughness, pore size, and mechanical properties for cell growth in soft tissue, as the mechanical properties of the scaffolds are rapidly reduced due to the brittleness of PHEMA during morphological modification.

Methyl methaceylate (MMA) is a versatile monomer used in various industrial fields, particularly in the production of polymethyl methacrylate (PMMA) via polymerization. PMMA is also widely used to create scaffolds in tissue engineering due to its many properties including mechanical strength, biocompatibility, porosity, transparency, and non-biodegradability. MMA scaffolds are used in bone [35,36,37] and cartilage [38,39] tissue engineering, in dental implants and prosthetics [40,41], for drug delivery [42,43], as materials for contact lenses and artificial corneas [44,45], and to promote the growth of various tissues such as skin, muscle, and nerve tissues [46,47,48]. Although PMMA scaffolds are used in tissue engineering, they have limitations such as a lack of bioactivity and difficulties in cell adhesion. To address these issues, research efforts have focused on surface coating with ECM, the incorporation of nanoparticles, and surface modification techniques [49,50,51].

A number of studies have explored the development of poly(HEMA-co-MMA) hydrogel scaffolds for tissue engineering applications as they exhibit advantageous properties. Yan et al. demonstrated the potential of surface modification through the attachment of biomolecules such as collagen and bFGF onto poly(HEMA-co-MMA) hydrogel scaffolds, facilitating cell adhesion [52]. Additionally, the development of high-strength nerve guidance channels [53] for central nervous system applications, bone regeneration [54,55], and drug delivery scaffolds for cancer therapy [56]. These studies highlight the versatility of poly(HEMA-co-MMA) hydrogels in tissue engineering, enabling the creation of biomimetic scaffolds that can mimic the extracellular matrix and promote tissue regeneration. While poly(HEMA-co-MMA) hydrogel scaffolds have shown promise in tissue engineering, these materials often require further modifications to enhance their bioactivity and mechanical properties. For example, to promote cell adhesion and proliferation, it is frequently necessary to functionalize the scaffold surface with bioactive molecules such as growth factors or extracellular matrix proteins [52]. Additionally, the development of high-strength scaffolds has primarily focused on applications in bone tissue engineering, limiting their potential for soft tissue regeneration.

In this study, we fabricated scaffolds suitable for soft tissue engineering using PHEMA and MMA. The polymerization process induced phase separation solely through self-assembly, allowing the modification of the surface structure, pore formation, and the achievement of various fabricated scaffolds by adjusting the PHEMA-to-MMA ratio without the addition of other biomaterials or biomolecules. The fabricated scaffolds demonstrated appropriate compressive strength and modulus, tensile strength and modulus, and biocompatibility for soft tissue applications. Furthermore, the effects of the poly(HEMA-co-MMA) scaffolds on the cell adhesion and proliferation were addressed using human dermal fibroblasts (HDFs).

## 2. Materials and Methods

### 2.1. Materials

All the reagents in this experiment were used without purification. The reagents used to prepare the hydrogel scaffolds were as follows: 2-hydroxyethyl methacrylate (HEMA; Sigma-Aldrich, St. Louis, MO, USA) and methyl methacrylate (MMA; Sigma-Aldrich, USA), used as monomers; dimethyl formamide (DMF; Sigma-Aldrich, USA) and distilled water (DW) as a solvent; and pentaerythritol tetraacrylate (PETA; Sigma-Aldrich, USA) as a crosslinking agent. As an initiator, 99% ammonium persulfate (APS; Sigma-Aldrich) was used, and 99% tetramethyl ethyl ethylene diamine (TEMED; Acros organics, Antwerpen, Belgium) was used as a catalyst. All reagents except DW were purchased from Sigma-Aldrich. DW was purified using a purified water purification system (Direct-Q^®^ Water Purification System, milli-Q, Darmstadt, Germany), and tertiary purified water was used.

### 2.2. Hydrogel Scaffold Preparation

#### 2.2.1. PHEMA Hydrogel Scaffold Preparation

**Figure 1:**
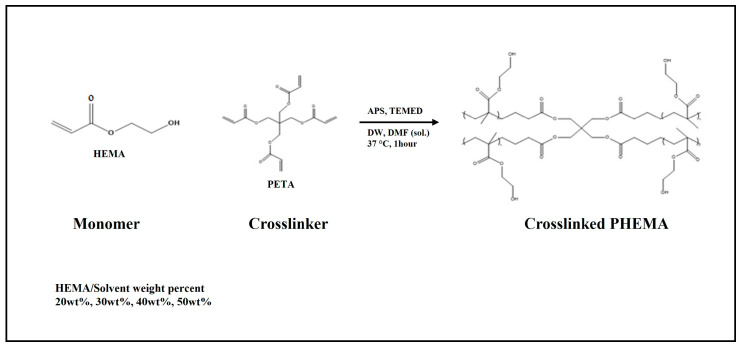
Polymerization mechanism of PHEMA hydrogel scaffold.

PHEMA hydrogel scaffolds were prepared to analyze their surface properties according to the concentration of HEMA and its physical properties. The samples of monomer concentrations with 50 wt%, 40 wt%, 30 wt%, and 20 wt% HEMA were denoted as HC50, HC40, HC30, and HC20, respectively. The ratio of solvent was fixed with DW/DMF = 80:20 (*w/w*), and the crosslinker PETA (2.8 mol%), initiator APS (3.0 mol%), and catalyst TEMED (3.0 mol%) were added to a solution considering the molar ratio of the total solution. Thereafter, the homogeneous solution was poured into Petri dishes (90 × 15 mm, SPL life science, Pocheon-si, Republic of Korea) and polymerized at 37 °C for 1 h. After confirming that the solution had transitioned into a gel state during the polymerization process, a hydrogel was obtained, and any unreacted residual monomers were removed by washing twice with ethanol and DW (Figure 1).

#### 2.2.2. Poly(HEMA-co-MMA) Hydrogel Scaffold Preparation

**Figure 2:**
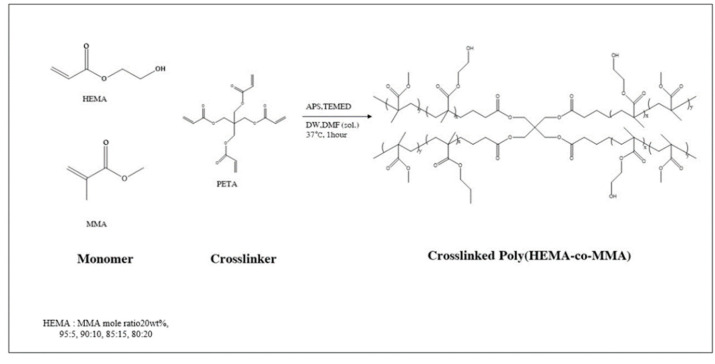
Polymerization mechanism for poly(HEMA-co-MMA) hydrogel scaffold.

A poly(HEMA-co-MMA) hydrogel scaffold was synthesized by selecting an optimal concentration of PHEMA. Several molar ratios of HEMA to MMA were prepared (95:5, 90:10, 85:15, 80:20). Here, the solvent and crosslinker were fixed at the same ratio as the PHEMA hydrogel scaffold. In addition, the initiator and the catalyst were selected at 6 mol% with the highest gel fraction value of poly(HEMA-co-MMA) hydrogel. The prepared comparative group was washed using the same method as the PHEMA hydrogel scaffold mentioned above to prepare the sample (Figure 2).

### 2.3. Analytical Methods

#### 2.3.1. Scanning Electron Microscope (SEM) and Pore Size Distribution

The prepared scaffolds were freeze-dried and observed using an SEM (ISP, IM-60, ChemKnock, Seoul, Republic of Korea) to compare the morphologies of the PHEMA and poly(HEMA-co-MMA) hydrogel scaffolds. Briefly, the scaffolds were cut into disks with a thickness of 5 mm and a diameter of 15 mm and freeze-dried. The dried scaffold samples were then fixed onto a stub and gold-coated before the SEM observations. In this case, the voltages were 10 KV and 15 KV, and all samples were observed at 3000 magnifications. The size distribution of the open pores on the surfaces of the PHEMA and poly(HEMA-co-MMA) hydrogel scaffolds was measured using ImageJ software version 1.54g (NIH, Bethesda, MD, USA). The size distribution was determined from at least 50 selected open pores in each SEM image.

#### 2.3.2. Fourier-Transform Infrared (FT-IR) Spectroscopy

Functional groups were analyzed using an FT-IR spectroscope (Rohde-Schwarz, Columbia, SC, USA) to confirm the copolymerization of the poly(HEMA-co-MMA). The sample was freeze-dried and milled into powder. The sample was measured using pellets with KBr. All measurements were taken 32 times, and the measurement range was set to 400–4000 cm^−1^.

#### 2.3.3. Mechanical Test

A universal testing machine (Instron 3345) was used to analyze the physical properties of the hydrogel. Samples were manufactured in cylindrical (diameter = 5 mm, height = 10 mm) and rectangular (width = 5 mm, length = 30 mm) shapes for compressive and tensile testing. The mechanical properties were measured at a speed of 5 mm/min. In the case of the tensile test, measurements were taken after fixing the sample by attaching sandpaper to the jig to prevent slipping during the measurement.

#### 2.3.4. Wettability

The contact angle (OCA20, Data Physics Instruments, Filderstadt, Germany) was measured to confirm the change in wettability according to the surface morphology and composition ratio. To measure the contact angle, a slide glass with solution was spin-coated and polymerized. The contact angles of the prepared specimens were measured at five points.

#### 2.3.5. Cell Culture and Cell Proliferation Assay

Fetal human dermal fibroblasts (HDFs) were purchased from Genlantis (San Diego, CA, USA). The HDFs were maintained at 37 °C in Dulbecco’s modified Eagle medium (DMEM, Welgene, Republic of Korea) containing 10% fetal bovine serum (FBS; Biological Industries, Göttingen, Germany) and 1% penicillin–streptomycin (PS; Gibco, Waltham, MA, USA). The culture medium was changed every three days, and the cells were passaged at sub-confluency. Then, 100,000 HDFs were seeded in each well of 12-well cell culture plates and cultured for 24 h. After 24 h, the culture medium was changed with 1 mL of DMEM and co-cultured with five different types of scaffolds using a cell culture insert (SPL lifescience, Pocheon-si, Republic of Korea). The cell proliferation activity was measured at 1, 3, and 7 days using an EZ-Cytox cell viability assay kit (DogenBio, Seoul, Republic of Korea). Briefly, the HDFs were washed with PBS and then treated with a mixture of 50 μL EZ-Cytox solution and 500 μL DMEM for 3 h in a cell culture incubator, following the manufacturer’s instructions. After incubation, the absorbance was analyzed at 450 nm using a microplate spectrophotometer (SpectraMAX Plus, Molecular Devices, San Jose, USA) (*n* = 3). To identify the effects of scaffolds on the HDFs, the co-cultured HDFs were fixed with 4% paraformaldehyde solution and stained with Alexa Fluor 488 Phalloidin (Thermo Fischer, Waltham, USA) for F-actin filament and DAPI (4′,6-diamidino-2-phenylindole) (Thermo Fisher, Waltham, USA) for the nucleus of the HDFs. The morphology of the stained HDFs was observed and analyzed using an Olympus fluorescence microscope (Olympus CKX53, Tokyo, Japan).

#### 2.3.6. Cell Adhesion Observation Via SEM

The HDF cells were fixed and dehydrated to confirm the shapes of the cell attachment in the poly(HEMA-co-MMA) hydrogel scaffold. Prior to cell fixation, the cells were washed twice with cold 0.1 M PBS to remove residual culture medium and serum debris. The cells were fixed in 0.1 mL of 2.5% glutaraldehyde in PBS overnight. After fixation, all residual fixative solutions were removed, and the cells were washed twice with cold PBS for 5 min. The fixed cells were treated with graded concentrations of ethanol for dehydration (30%, 50%, 75%, 95%, 100%). The dehydration solution was added every 5 min, and the samples were immediately lyophilized using a freeze-dryer (LP-10, IlshinBiobase, Seoul, Republic of Korea) to minimize the degradation of the hydrogel scaffolds.

#### 2.3.7. Statistical Analysis

GraphPad Prism version 7.00 (GraphPad, Boston, MA, USA) was used to perform all the statistical analyses. The data were expressed as the mean ± standard deviation from at least three independent experiments. Statistical significance was analyzed via one-way analysis of variance (ANOVA) followed by a Tukey–Kramer post hoc test. A value of *p* < 0.05 was considered to be statistically significant (* *p* < 0.05, ** *p* < 0.01, and *** *p* < 0.001). A graphical representation of all data was created using Origin 8.0 software (Origin Lab, Northampton, MA, USA).

## 3. Results and Discussion

### 3.1. Comparison of PHEMA Hydrogel Scaffold Morphology According to HEMA Concentration

**Figure 3:**
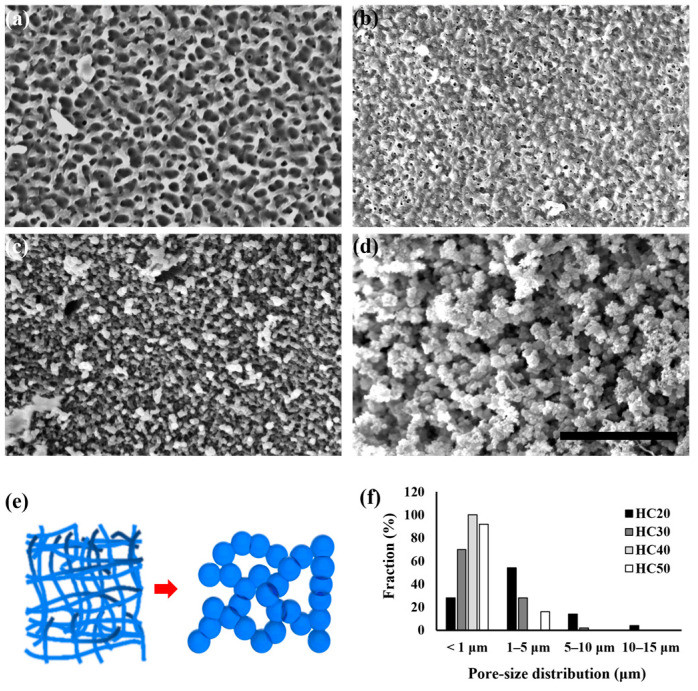
Representative SEM micrographs of (**a**) HC50, (**b**) HC40, (**c**) HC30, and (**d**) HC20. The schematic diagram in (**e**) shows the morphological changes in the scaffolds depending on the proportion of HEMA. (**f**) The pore-size distribution of scaffolds. Scale bar indicates 50 μm.

Figure 3 shows the morphology of the PHEMA hydrogel according to the concentration of HEMA. In the SEM images shown in Figure 3a–d, all the surface morphologies were changed when the concentration of HEMA decreased and the solvent increased. In more detail, HC50 exhibited closed, nano-sized pores with a uniform shape. In the case of HC40 and HC30, the size of the pores with irregular shapes became smaller. In HC30, it can be seen that the pores were bead-shaped. The micro-pore size and bead shape could also be observed by the agglomeration of the polymer chain in HC20. This phenomenon could be explained by the properties of PHEMA with its hydrophilic and hydrophobic functional groups. When the ratio of the polymer was high, linear polymer chain characteristics were exhibited. However, when the solvent concentration increased, an agglomerated polymer chain was exhibited instead, as shown in Figure 3e. The increased concentration of PHEMA molecules led to a decrease in solvent–polymer interactions, resulting in an increase in polymer–polymer interactions and subsequent aggregation [57]. Therefore, various morphologies were observed according to the concentration. Figure 3f shows the pore size distribution of the PHEMA hydrogel according to the concentration of HEMA. The average pore diameter of the HC20, HC30, HC40, and HC50 scaffolds were 2.93 μm, 1.06 μm, 0.17 μm, and 0.49 μm, respectively.

### 3.2. Evaluation of PHEMA Hydrogel Scaffold Characteristics According to HEMA Concentration

**Figure 4:**
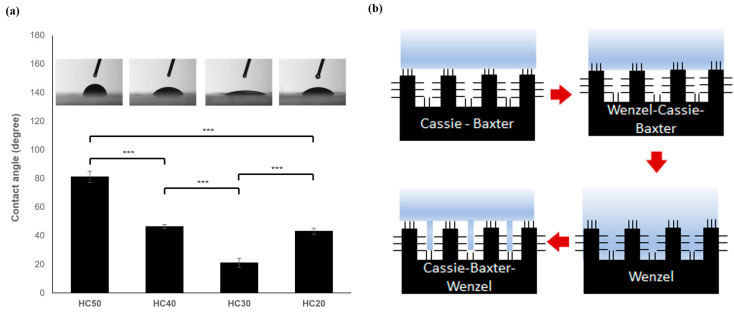
(**a**) Change in contact angle according to HEMA concentration. (**b**) Cause of contact angle change analyzed using the Wenzel and Cassie–Baxter models (*** *p* < 0.001).

Figure 4 shows the contact angles on the surfaces of the PHEMA hydrogel scaffolds using the Wenzel and Cassie–Baxter models according to the concentration. As shown in Figure 4a, the contact angle according to the concentration was found to be 81.2 ± 4.6° (HC50), 46.4 ± 2.3° (HC40), 21.1 ± 1.6° (HC30), and 43.2 ± 2.2° (HC20), respectively. In terms of the analysis using the Wenzel and Cassie–Baxter models, as shown in Figure 4b, HC50 appears to be unaffected by the micro/nanostructure because it is homogeneous and has closed pores, as shown in Figure 3a. The Wenzel–Cassie–Baxter model properties for HC40 show that it is affected by nanostructured surfaces, as the agglomeration of the polymer chain begins to occur. HC30 exhibits nano- and micro-pores as the agglomeration progresses and is the most hydrophilic according to the Wenzel model. Moreover, the hydrophobic properties of HC20 increased, and it can be seen by the Wenzel–Cassie–Baxter model that this sample was affected by the microstructure as the phase separation intensified. Phase separation is a phenomenon where a homogeneous mixture of two or more components separates into distinct phases, each with different properties. In the context of polymers, phase separation often occurs when a polymer solution becomes unstable due to changes in temperature, solvent composition, polymer concentration, molecular weight, polymer architecture, or other factors. This phase separation is exploited to create polymer blends, induce self-assembly, and enable controlled release functionalities [58,59,60,61,62]. As such, the change in the surface shape that can be seen in Figure 4 indicates that the surface property may be freely adjusted even when the same materials are used, allowing it to be used for various purposes such as inducing cell detachment and attachment using different HEMA concentration ratios.

**Figure 5:**
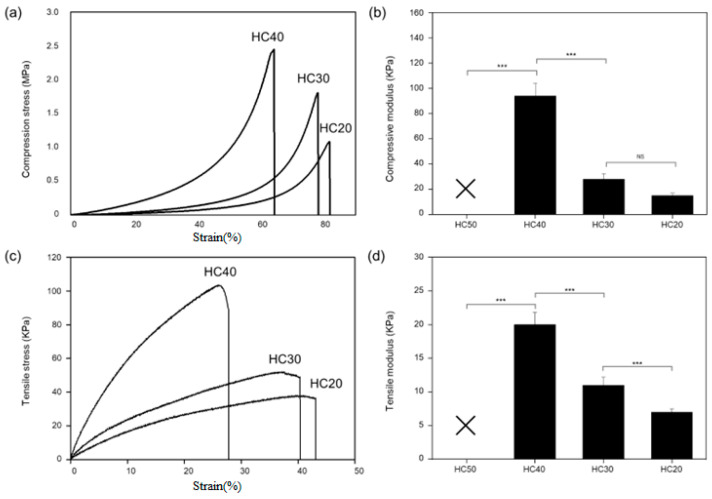
Change in the physical properties of HEMA hydrogel scaffold. (**a**) Compressive stress–strain curve, (**b**) compressive modulus, (**c**) tensile stress–strain curve, and (**d**) tensile modulus of HEMA hydrogel scaffolds (NS: nonsignificant, *** *p* < 0.001).

Figure 5 shows the tensile strength and compressive strength of the PHEMA hydrogel scaffolds. In this case, HC50 could not be measured because cracks occurred during the mechanical experiment. As shown in Figure 5a,b, the values of compressive strength and modulus for HC40, HC30, and HC20 were measured as 2.45 ± 0.42 MPa/94 ± 4.71 KPa, 1.80 ± 0.21 MPa/28.41 ± 1.42 KPa, and 1.08 ± 0.34 MPa, 15.14 ± 0.75 KPa, respectively. This result shows that as the concentration of HEMA increases, the mechanical characteristics of the hydrogel become stronger. Likewise, in the tensile S-S curve and modulus shown in Figure 5c,d, the tensile strength decreased as the polymer concentration decreased, as in the compressive S-S curve. As shown in the SEM data in Figure 3, this phenomenon is due to the weakening of the physical properties that occur as the bonding force of the polymer chain is weakened by phase separation.

### 3.3. Assessment of Poly(HEMA-co-MMA) Hydrogel Scaffold Characteristics According to MMA Concentration

PHEMA hydrogel is limited in its use as a scaffold because its surface wettability and mechanical properties are inversely proportional depending on the polymer concentration. In this research, poly(HEMA-co-MMA) hydrogel scaffolds were manufactured using MMA with hydrophobic properties to overcome the limitations of the PHEMA hydrogel scaffold. Here, PETA was employed as a crosslinking agent for the copolymerization of PHEMA and MMA to create a denser crosslinked network, thereby achieving mechanical properties and porosity suitable for soft tissue. The multiple functional groups of PETA facilitated the formation of a more interconnected network structure [63,64].

**Figure 6:**
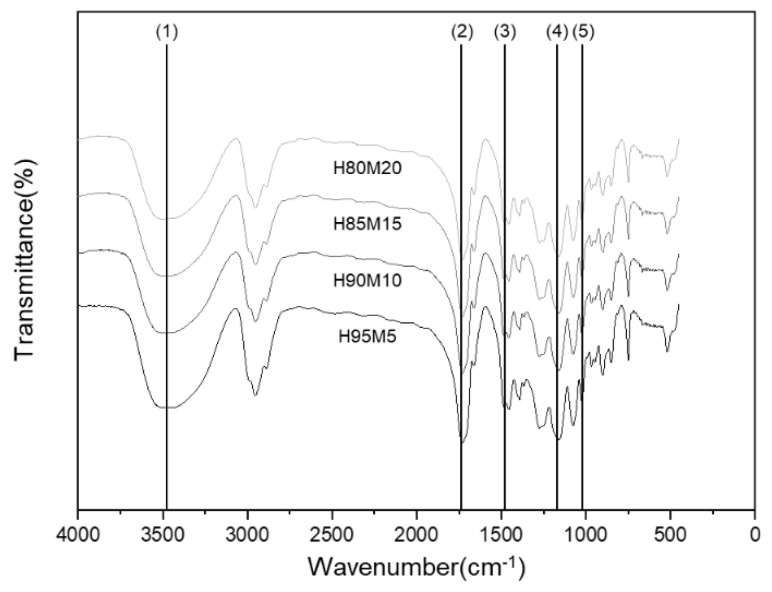
FT-IR spectra of poly(HEMA-co-MMA) according to molar ratio of HEMA and MMA monomer.

Experiments were conducted on the manufactured poly(HEMA-co-MMA) hydrogel scaffold with the aim of simultaneously increasing its wettability and mechanical properties. In the preparation of the poly(HEMA-co-MMA) hydrogel scaffold, a 30 wt% monomer concentration was used to provide superior wettability compared to the PHEMA hydrogel scaffolds. As illustrated in Figure 6, FT-IR analysis was performed to determine the copolymerization of the poly(HEMA-co-MMA) hydrogel. The results of the FT-IR analysis show that the functional groups of HEMA and MMA are similar, so the overall peak form is also similar. However, when the HEMA content changes, the peak intensity ratios of the hydroxyl group (3463 cm^−1^), carbonyl (1734 cm^−1^), and carboxylic acid (1475 cm^−1^, 1166 cm^−1^) also change because of the hydroxyl group that is only present in HEMA (Table 1). Thus, it can be verified that the polymerization was successful according to the HEMA and MMA ratios [3].

**Figure 7:**
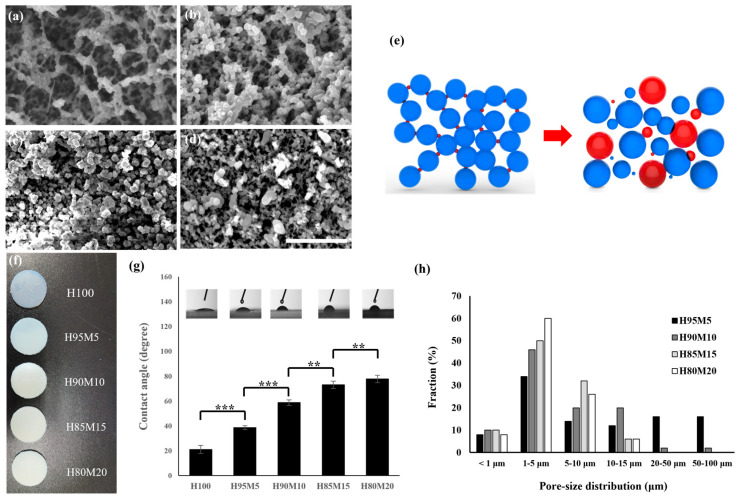
Surface morphology and surface characteristics according to the ratio of HEMA and MMA. SEM images of (**a**) H95M5, (**b**) H90M10, (**c**) H85M15, and (**d**) H80M20. (**e**) Scheme of morphological change from H95M5 to H80M20. (**f**) Photograph of transparency according to the molar ratio of HEMA and MMA. (**g**) Static water contact angle of poly(HEMA-co-MMA). (**h**) Pore size distribution of poly(HEMA-co-MMA). Scale bar indicates 20 μm (** *p* < 0.01, *** *p* < 0.001).

Figure 7 shows the results of the analysis of the poly(HEMA-co-MMA) hydrogel scaffold surface characteristics according to the various ratios of HEMA and MMA. Figure 7a–d shows the morphologies of the poly(HEMA-co-MMA) hydrogel scaffold according to the HEMA and MMA ratios. Figure 7a,b shows that the molar ratio of HEMA and MMA was less than 90:10 and that the agglomeration polymer chains were connected. When this was compared with the PHEMA hydrogel scaffold in Figure 3d, the PHEMA hydrogel scaffold showed an agglomerated morphology. However, the poly(HEMA-co-MMA) hydrogel scaffold had a large inter-chain coupling effect between the agglomeration chains of HEMA due to the MMA, as depicted in Figure 7a,e. As shown in Figure 7c,d, when the amount of MMA increased, phase separation occurred due to the hydrophilic and hydrophobic properties, resulting in a wider gap between the agglomeration particles and an irregularity in the agglomerated particle size. Consequently, when the content of MMA decreases below a certain concentration, the PMMA can act as a second crosslinker for the PHEMA due to the hydrogen bonding activities of the acrylic acid and hydroxyl group, but when the MMA content increases above a certain concentration, it causes rapid phase separation (Figure 7e). In the photo shown in Figure 7f, it can be seen that as the content of MMA increased, the agglomerated particle size became irregular due to phase separation, an indication that transparency reduced and the sample became opaquer. In terms of the contact angle shown in Figure 7g, the contact angles of the poly(HEMA-co-MMA) hydrogel scaffolds were found to be 36.9 ± 1.8° (H95M5), 57.0 ± 2.7° (H95M10), 65.9 ± 3.4° (H85M15), and 74.7 ± 3.7° (H80M20), respectively. The results show that as the PMMA content increases, hydrophobic properties are manifested according to the concentration ratio in the poly(HEMA-co-MMA) hydrogel scaffold. Although hydrophobic MMA is added, the wettability characteristics of the poly(HEMA-co-MMA) hydrogel scaffold were better than those of the HC50 hydrogel scaffold consisting of only HEMA; this was due to the morphology characteristics of the poly(HEMA-co-MMA) hydrogel scaffold. Figure 7h shows the pore size distribution for the poly(HEMA-co-MMA) hydrogel scaffolds. The average diameters of the pores were 23.19 μm (H95M5), 7.35 μm (H95M10), 4.67 μm (H85M15), and 4.64 μm (H80M20). The pore size of the scaffolds was predominantly within the 1–80 μm range. Figure 7 demonstrates that it is possible to manufacture poly(HEMA-co-MMA) hydrogel scaffolds with a morphology that has better wettability than that using HEMA alone; this is because of the additional interactions that occur when an appropriate amount of MMA is injected.

**Figure 8:**
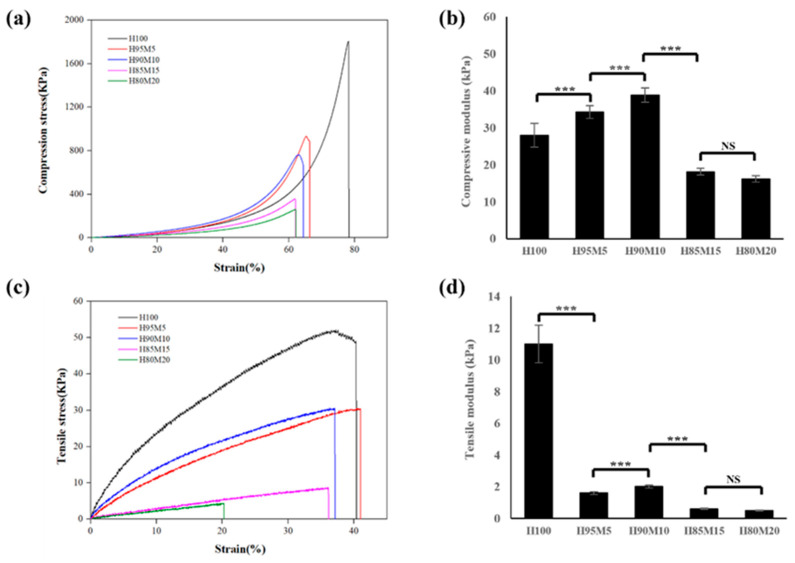
Change in mechanical properties of poly(HEMA-co-MMA). (**a**) Compressive stress–strain curve, (**b**) compressive modulus, (**c**) tensile stress–strain curve, and (**d**) tensile modulus of poly(HEMA-co-MMA) hydrogel scaffolds (NS: nonsignificant, *** *p* < 0.001).

In terms of the mechanical properties of the poly(HEMA-co-MMA) hydrogel scaffold according to the HEMA and MMA ratios, the values of compressive strength and modulus were 932.4 ± 52.5 KPa/34.2 ± 1.7 KPa (H95M5), 761.2 ± 24.2 KPa/38.8 ± 1.9 KPa (H90M10), 355.2 ± 50.4 KPa/18.1 ± 1.0 KPa(H85M15), and 254.2 ± 36.4 KPa/16.1 ± 0.8 KPa (H80M20), respectively (Figure 8a,b). Moreover, the tensile strength and modulus were 30.5 ± 2.1 KPa/1.6 ± 0.1 KPa (H95M5), 30.6 ± 3.7 KPa/2.0 ± 0.1 KPa (H90M10), 8.74 ± 1.50 KPa/0.60 ± 0.03 KPa (H85M15), and 4.4 ± 1.3 KPa/0.5 ± 0.1 KPa (H80M20), respectively (Figure 8c,d). From the compressive strength and modulus measurements, it was confirmed that H95M5 and H90M10 exhibited better mechanical properties than HC30 due to the interaction of HEMA and MMA. However, the mechanical properties of H85M15 and H80M20 exhibited a sharp decline, as shown in Figure 8. This phenomenon is assumed to be caused by immiscibility as the amount of hydrophobic MMA increases. Similarly, in the tensile test shown in Figure 8c,d, the mechanical properties increased to H90M10, and then the properties decreased sharply from H85M15. This result verified the fact that the mechanical properties and morphology of poly(HEMA-co-MMA) hydrogel scaffolds at certain concentrations were better than those of PHEMA hydrogel scaffolds.

### 3.4. Biocompatibility of Poly(HEMA-co-MMA) Hydrogel Scaffolds with Various Concentrations of HEMA and MMA

**Figure 9:**
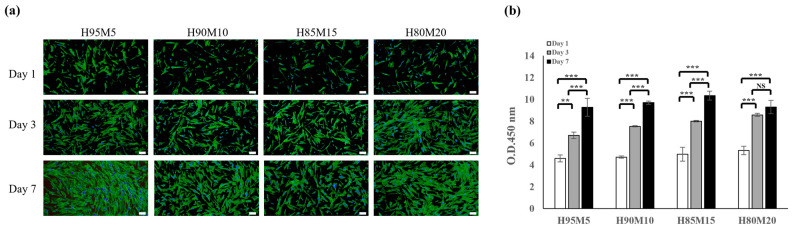
Biocompatibility of poly(HEMA-co-MMA) hydrogel. (**a**) Confocal images of cell adhesion on poly(HEMA-co-MMA) hydrogel scaffolds. The nucleus was stained with DAPI (blue) and the F-actin filament of cytoplasm were stained with Alexa Fluor 488 Phalloidin (green); (**b**) cell proliferation assay. Scale bar indicates 200 μm (NS: nonsignificant, ** *p* < 0.01, *** *p* < 0.001).

Figure 9 shows the biocompatibility of the poly(HEMA-co-MMA) hydrogel scaffolds. Cell viability and cell proliferation assays were performed using fetal human dermal fibroblasts (HDFs). The HDFs were co-cultured with the scaffolds for 1, 3, and 7 days. The co-cultured HDFs were highly viable under the experimental conditions and did not show any significant cell toxicity in any of the scaffold groups (Figure 9). The morphology of the HDFs under scaffold co-culture conditions demonstrated a well-extended cytoskeleton (Figure 9a) and proliferation (Figure 9b). Therefore, we developed poly(HEMA-co-MMA) hydrogel scaffolds with enhanced mechanical properties, wettability, and biocompatibility compared with PHEMA hydrogel scaffolds.

### 3.5. Cell Adhesion and Morphology of Poly(HEMA-co-MMA) Hydrogel Scaffolds with Various Concentrations of HEMA and MMA

**Figure 10:**
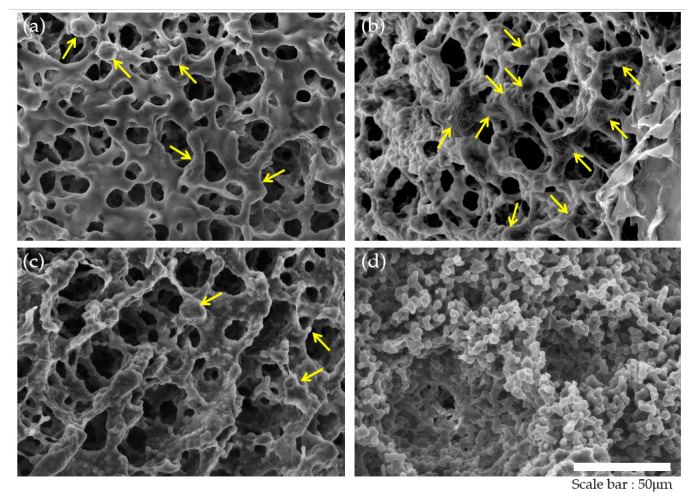
SEM images of human dermal fibroblasts (HDFs) attached to poly(HEMA-co-MMA) hydrogel scaffolds: (**a**) H95M5, (**b**) H90M10, (**c**) H85M15, and (**d**) H80M20. Arrows indicate the attached HDFs on the scaffold on day 1. Scale bar indicates 50 μm.

The HDF cell adhesion and morphology in the poly(HEMA-co-MMA) hydrogel scaffolds were observed using SEM analysis. Cells were incubated on scaffolds under different conditions in H95M5, H90M10, H85M15, and H80M20 for a day, and immediately fixed and dehydrated. Prior to the SEM observations, the HDF cells attached to the scaffolds were lyophilized and imaged under a 750× magnification SEM to analyze the cell adherence and morphology for each condition. Figure 10a–c represents the adhesion and spreading of the cells on the surfaces of H95M5, H90M10, and H85M15 hydrogel scaffolds. These morphologies showed that the surfaces and pore sizes of the scaffolds provided a biocompatible structure for cell adhesion and proliferation. As can be seen from Figure 10d, even though the H80M20 hydrogel scaffold was a non-cytotoxic substance, it was confirmed that the HDF cells could not attach to the scaffolds. This is because the irregular surface and small pore sizes made it a difficult environment for cells to adhere to and grow in. According to Paterson et al., the hydroxyl-rich surface of PHEMA makes it non-adhesive to living cells. Therefore, to utilize PHEMA scaffolds for tissue engineering, attempts have been made to overcome this limitation by incorporating or functionalizing carboxyl and sulfonyl groups, but these approaches have not been highly effective [65]. Based on the results of this study, we confirmed that the poly(HEMA-co-MMA) hydrogel scaffolds controlled by the proper ratio of HEMA and MMA successfully produced an optimal scaffold that allowed for the adhesion and proliferation of cells.

## 4. Conclusions

In this study, to overcome the limitations of synthetic hydrogel scaffolds that only consist of PHEMA, a synthetic hydrogel scaffold fabricated through the copolymerization of HEMA and MMA was proposed. For soft tissue applications, hydrogel scaffolds require suitable biocompatibility and mechanical properties, roughness, pore size, and interconnectivity among pores. Therefore, to investigate the properties of the proposed poly(HEMA-co-MMA) hydrogel scaffold, the morphology, wettability, and biocompatibility were evaluated according to various concentrations of HEMA and MMA, and were compared with PHEMA hydrogel scaffolds. Consequently, we determined the most suitable concentration of HEMA and MMA that can be used to fabricate synthetic hydrogel scaffolds with better mechanical properties and biocompatibility than PHEMA hydrogel scaffolds, achieved through hydrophilic–hydrophobic interactions and hydrogen bonding. Moreover, the proposed method can fabricate poly(HEMA-co-MMA) hydrogel scaffolds that can enable cell adhesion or non-adhesion through various surface morphologies created using the phase separation of HEMA and MMA.

## Figures and Tables

**Table 1:** Peak table for FT- IR spectra of poly(HEMA−co−MMA).

Peak No.	Wavelength (cm^−1^)	Functional Group
1	3463	−OH
2	2944	SP^3^ Carbon
3	1734	C = O
4	1475	−COO_sym_
5	1166	−COO_asym_

## Data Availability

The experimental data from this study are available upon request.
